# Silver nanoparticles combine with Nigella sativa oil to potentiate apoptosis in cervical cancer

**DOI:** 10.1038/s41598-026-36082-4

**Published:** 2026-04-07

**Authors:** Kobra Hosseini, Mahla Alizadeh, Azadeh Jalilian, Sedighe Hoseini, Soroush Bahrami, Atefeh Shamosi, Alireza Shams, Mahin Seifi Alan, Ali Hashemi, Parviz fallah, Soheila Madadi

**Affiliations:** 1 Clinical Research and Development Center of the Kamali Hospital, Alborz University of Medical Sciences, Karaj, Iran; 2 Non-Communicable Disease Research Center of Tabatabaei, Alborz University of Medical Science, Karaj, Iran; 3 Department of Biology, University of Science and Culture, Tehran, Iran; 4 Department of Biology, Faculty of Science, Shahid Bahonar University of Kerman, Kerman, Iran; 5 Cardiovascular Research Center, Alborz University of Medical Sciences, Karaj, Iran; 6 Department of Anatomy, School of Medicine, Alborz University of Medical Science, Karaj, Iran; 7 Department of Pathology, School of Medicine, Alborz University of Medical Science, Karaj, Iran; 8 Checkup Clinical and Speciality Laboratory, Karaj, Iran; 9 Laboratory Science Department, Allied Medicine Faculty, Alborz University of Medical Sciences, Karaj, Iran

**Keywords:** Cervical cancer, Nigella sativa, Silver nanoparticles, Synergistic apoptosis, Oxidative stress, Cancer, Cell biology

## Abstract

Cervical cancer remains a global health challenge, necessitating innovative therapies that enhance tumor-specific apoptosis while sparing healthy tissues. This study investigates the combined potential of Nigella sativa oil (NS) and silver nanoparticles (AgNPs) in inducing apoptosis in HeLa cervical cancer cells. NS, rich in thymoquinone, modulates apoptotic pathways (Bax/Bcl-2, p53, caspase-9), while AgNPs trigger oxidative stress and genomic instability. We hypothesized that their combination would amplify apoptotic efficacy through complementary mechanisms. HeLa cells were treated with AgNPs (1–10 µg/mL) and with a combination of a selected dose of AgNPs (5 µg/mL) and NS (100–250 µg/mL). Cytotoxicity, apoptosis (as assessed by Annexin V/PI staining and acridine orange/ethidium bromide staining), and gene expression (Quantitative Real-Time Polymerase Chain Reaction, qRT-PCR) were evaluated. Gas Chromatography-Mass Spectrometry (GC-MS) confirmed NS’s bioactive composition, while Scanning Electron Microscopy (SEM)/Fourier Transform Infrared Spectroscopy (FTIR) characterized AgNPs. The results demonstrated dose-dependent apoptosis. The most effective combination was 5 µg/mL AgNPs + 150 µg/mL NS, which induced 29.1% early apoptosis. Pro-apoptotic genes (BAX, BAK, p53, caspase-9) were upregulated (3.2–4.8-fold), while BCL-2 was suppressed (0.28-fold). This study unveils a novel combinatorial strategy leveraging NS’s epigenetic regulation and AgNPs’ cytotoxic effects, suggesting a promising avenue for future research. The findings underscore the potential of plant-nanoparticle synergy in oncology. Future in vivo studies are warranted to validate the clinical applicability.

**Supplementary Information:**

The online version contains supplementary material available at 10.1038/s41598-026-36082-4.

## Introduction

Cervical cancer continues to be a leading cause of cancer-related deaths globally. Without expanded prevention efforts, its burden is projected to rise^1^. Although treatment advancements have been made, cervical cancer remains incurable in many cases worldwide^2^. This underscores the urgent need for targeted, innovative therapies that enhance tumor control while minimizing damage to healthy tissues. Apoptosis, or programmed cell death, is essential for maintaining cellular homeostasis^3^. Cancer is characterized by the deregulation of these apoptosis pathways, which allows tumor cells to evade death despite uncontrolled proliferation, thereby promoting their long-term survival^4^. Apoptotic mediators like caspase-9, Bax, Bak, and p53 induce cell death, while anti-apoptotic proteins like Bcl-2 inhibit it. Reestablishing the apoptotic pathways in cancer cells is a viable therapeutic approach^5^.

Recent advances in nanotechnology have introduced new possibilities for cancer treatment^6^. Silver nanoparticles (AgNPs) exhibit potent anticancer activity through mechanisms like oxidative stress, interference with mitochondrial membrane function, and induction of DNA damage^7^. Their nanoscale size and high surface-to-volume ratio enable them to interact specifically with cellular constituents, making them ideal agents for targeted therapy. However, concerns regarding nanoparticle-induced oxidative stress and cytotoxicity against normal cells necessitate enhancing their selectivity and efficacy^8^.

Natural compounds like Nigella sativa oil (NS) have been highlighted for their multi-functional anticancer effects. NS is abundant in thymoquinone (TQ), which regulates apoptotic signaling, suppresses inflammation, and reduces oxidative stress. Evidence indicates that it can suppress anti-apoptotic Bcl-2 and promote pro-apoptotic Bax and caspase-9, thereby inducing cell death in various cancer models. Furthermore, NS’s antioxidant action may counteract AgNPs’ oxidative damage, yielding a synergistic therapeutic effect.

Based on their established mechanisms reported in the literature, AgNPs kill cancer cells through direct oxidative stress and genomic damage, while NS may complement this by regulating pro-apoptotic gene expression as well as by scavenging Reactive Oxygen Species (ROS)^7,8^. Synergy can counteract the drawbacks of monotherapies due to complementary modes of action. This study is the first to investigate the simultaneous combination effects of AgNPs and NS in cervical cancer cells. Hence, the synergism of NS and AgNPs may offer a novel approach for achieving maximum apoptosis induction.

## Materials

### Study design and variables

This experimental study aimed to investigate the effects of AgNPs and the combination of AgNPs with NS on the HeLa cancer cell line, a model of human cervical cancer. The experimental groups included:^1^ untreated control^2^, vehicle control (0.1% DMSO)^3^, AgNPs alone (1, 5, 10 µg/mL), and^4^ a fixed dose of AgNPs (5 µg/mL) combined with NS (100, 150, 200, 250 µg/mL). Groups with NS treatment alone were not included, as the primary objective was to evaluate the synergistic potential of the combination therapy.

In this study, the HeLa cell line was obtained from the Pasteur Institute of Tehran. At the beginning of the experiments, the cell line was verified to be free from microbial and viral contamination. All experiments were performed in the Cellular Culture Laboratory, Department of Anatomical Sciences, Faculty of Medicine, Alborz University of Medical Sciences.

### Data collection and study execution

#### Cell culture

HeLa cells were purchased from the Pasteur Institute Research Center. Cells were detached using enzymatic and mechanical methods and suspended in DMEM (Dulbecco’s Modified Eagle Medium) supplemented with 10% Fetal Bovine Serum (FBS). The cell suspension was incubated at 37 °C in a humidified atmosphere with 5% CO₂. The culture medium was replaced every three days, and cell growth was monitored daily using an inverted microscope. After four days, cells were harvested at 80–90% confluency by trypsinization, washed, and centrifuged at 2000 rpm for 5 min, then counted and seeded into corresponding cell culture plates for further experiments.

#### Extraction of NS

Fifty grams of the NS were ground with a mechanical grinder and mixed with 200 mL of 70% high-performance liquid chromatography (HPLC) grade ethanol to prepare the NS extract. It was then stirred on a magnetic stirrer for 48 h. The solution was rotary evaporated to remove the solvent and obtain an oil extract. The extract was stored at 4 °C until further use.

#### Synthesis of silver nanoparticles

AgNPs were synthesized using a chemical reduction method. Briefly, 30 mL of double-distilled water was placed in a sterile Erlenmeyer flask and cooled in an ice bath. Sodium borohydride (67 µL of 2 mM solution) was added, followed by the dropwise addition of 1 mL of 2 mM silver nitrate solution. The mixture was then combined with 20 mL of 2 mM trisodium citrate solution and centrifuged at 15,000 rpm for 20 min. The resulting nanoparticle pellet was washed three times with sterile distilled water to remove impurities, freeze-dried, and stored as a powder.

### Characterization of AgNPs

#### Fourier transform infrared spectroscopy (FTIR)

The synthesized AgNPs were characterized using FTIR spectroscopy (Thermo-Nicolet) in the range of 4000 –600 cm-1. The nanoparticles were mixed with potassium bromide to form a pellet, which was analyzed to obtain the infrared spectrum. Data processing was placed on a substrate and performed using Bruker OPUS software.

#### Scanning electron microscopy (SEM)

The morphology of the silver nanoparticles was examined using a Philips XL30 scanning electron microscope. The nanoparticle suspension was sonicated at 50 Hz for 30 min to prevent aggregation. A 10 µL aliquot of the suspension was dried and imaged under the SEM at Amirkabir University of Technology.

#### Gas chromatography-mass spectrometry (GC-MS) analysis

The chemical composition of the NS extract was analyzed using an Agilent GC-MS system. Approximately 2 µL of the extract was injected into the GC-MS apparatus equipped with a 70 eV electron ionization source. The analysis was conducted over 50 min, during which the separated compounds were detected and identified by comparing their mass spectra with reference data from the National Institute of Standards and Technology (NIST) library. Helium was used as the carrier gas at a flow rate of 1 mL/min, and the column temperature was maintained at 100 °C.

#### Treatment of HeLa cells

HeLa cells were seeded at 5 × 10^4^ cells per well in a 12-well tissue culture plate. For treatments involving NS, a stock solution was prepared in dimethyl sulfoxide (DMSO) and subsequently diluted in culture medium to achieve the desired concentrations, ensuring the final DMSO concentration did not exceed 0.1% (v/v). Cells were treated with various concentrations of AgNPs (1, 5, and 10 µg/mL) for 48 h. Based on flow cytometry results showing the highest early apoptosis and the lowest late apoptosis and necrosis, the optimal AgNP concentration (5 µg/mL) was selected. This concentration was then combined with different doses of NS extract (100, 150, 200, and 250 µg/mL) for another 48-hour treatment period. Cell viability and apoptosis were assessed using the following techniques.

#### Flow cytometry analysis

The level of apoptosis was measured by Annexin V-FITC and propidium iodide (PI) staining. Cells were trypsinized, washed with PBS, and resuspended in 1X Annexin V Binding Buffer. The samples were then analyzed using a BD FACSCalibur flow cytometer after staining with a mixture of Annexin V-FITC and PI, according to the manufacturer’s instructions. Data were analyzed with FlowJo software (version X, Tree Star Inc.) to determine the percentage of cells in early apoptosis, late apoptosis, and necrosis.

### MTT assay

Cell viability was assessed with the MTT(3-(4,5-dimethylthiazol-2-yl)-2,5-diphenyltetrazolium Bromide) assay, which is based on the reduction of MTT to formazan crystals by the mitochondrial reductase enzymes present in living cells. The hydrophobic NS extract was dissolved in 100% dimethyl sulfoxide (DMSO) to prepare a stock solution. This stock was then diluted in the cell culture medium such that the final concentration of DMSO in all treatment wells containing NS, as well as in the corresponding vehicle control wells, was 0.1% (v/v). For the MTT assay, the following control groups were included for accurate baseline measurement: a negative control (cells with fresh medium only), a vehicle control (cells with medium containing 0.1% DMSO), and a blank control (medium with 0.1% DMSO, without cells). Cell viability was calculated using the following formula:

% Viability = [(OD_drug_treated - OD_blank) / (OD_vehicle_control - OD_blank)] × 100.

Cells were plated in wells and, after treatment, were incubated with 0.5 mg/mL MTT solution for 4 h. Formazan crystals were solubilized in 400 µL of dimethyl sulfoxide (DMSO), the solution was transferred to a 96-well microplate, and the absorbance was measured at 570 nm using an ELISA plate reader.

### Acridine Orange-Ethidium bromide staining

Nuclear morphology was also examined by acridine orange-ethidium bromide staining. The cells were treated with the full range of concentrations as indicated in the results (AgNPs at 5 µg/mL, NS at 100–250 µg/mL, and their combinations). After treatment, cells were stained with acridine orange and ethidium bromide. To obtain quantitative data, apoptotic and necrotic cells were manually counted in multiple random fields under a fluorescence microscope. Qualitative analysis was also performed to visualize the nuclear morphology of apoptotic cells.

### Expression analysis

Total RNA was extracted from control and silver and NS treated HeLa cells using the CinnaPure RNA kit (SinaGen, IRAN). The quantity and purity of the RNA were evaluated by a NanoDrop instrument (Thermo Scientific, USA), and the integrity of the RNA was verified by 1% agarose gel electrophoresis. The concentration and purity of the RNA were evaluated by using a NanoDrop ND-1000 spectrophotometer (Thermo Scientific, USA), and the RNA integrity was confirmed by 1% agarose gel electrophoresis. Complementary DNA (cDNA) was synthesized using the FIREScript RT cDNA Synthesis Kit (Solis BioDyne, Estonia). Five genes (BAX, BAK, P53, CASPASE-9, and BCL-2) that are known as key regulators of apoptosis were selected, and the GAPDH gene was included as a reference gene for expression. Relative expressions of genes were evaluated in controls and silver-treated HeLa cells by utilizing RealQ Plus 2 × PCR Master Mix cyber-Green high ROX PCR Master Mix (Amplicon, Odense, Denmark) in Step One Plus Real-Time PCR equipment (QIAGEN Rotor-Gene Q real-time PCR, USA). The primer sequences are illustrated in Supplementary Table 1. Ethical Considerations.

The ethics statement is consistent with guidelines, and only the minimum number of cell samples necessary for statistical significance was used. All data will be published with affiliation with Alborz University of Medical Sciences (IR. ABZUMS. REC. 1396 155).

### Statistical analysis

All the experiments were performed in triplicate, and data were expressed as mean ± standard deviation (Mean ± SD). SPSS software (version 18) was used for statistical analysis (one-way ANOVA was used for significance). Statistical significance was defined at *p* < 0.05.

## Results

### Culturing of HeLa cancer cells

HeLa cells adhered to the culture flask within two days. By day five, the cells had reached 90% confluence, at which point they were subcultured to prevent over-confluence and subsequent cell detachment or death. On the second day, when the cell density was low, the cells exhibited a spindle-shaped, elongated morphology with pseudopodia (Supplementary Fig. 1A). At 95% confluence, the cells exhibited a polygonal morphology (Supplementary Fig. 1B).

### (GC-MS) analysis of Nigella sativa extract

The GC-MS analysis of the NS extract revealed a complex profile with 26 distinct peaks, indicating a rich mixture of bioactive compounds (Supplementary Fig. 2). The identification and relative quantification of the major constituents are summarized in Supplementary Table 2. The analysis confirmed the extract was predominantly composed of fatty acids, with linoleic acid (36.17%) and hexadecanoic acid (17.82%) identified as the most abundant components. Other notable compounds included eicosadienoic acid (10.91%), stearic acid (8.42%), and o-cymene (3.23%). This composition, particularly the high concentration of unsaturated fatty acids, is consistent with the reported bioactive profile of NS.

### (FTIR) spectroscopy of nanoparticles

FTIR spectroscopy of AgNPs synthesized from silver nitrate solution revealed broad peaks corresponding to various functional groups (Supplementary Table 3). The FTIR spectrum (Supplementary Fig. 3) confirmed the successful synthesis of AgNPs using this method.

### (SEM) of silver nanoparticles

Chemically synthesized AgNPs were characterized by SEM. The particles appeared granular upon deposition on the microscope grid at room temperature, but upon resuspension in the culture medium, the granulation was reduced, and well-separated particles were obtained. The average particle diameter was approximately 100 nm (Supplementary Fig. 4).

### Annexin V/PI flow cytometry

**Fig. 1:**
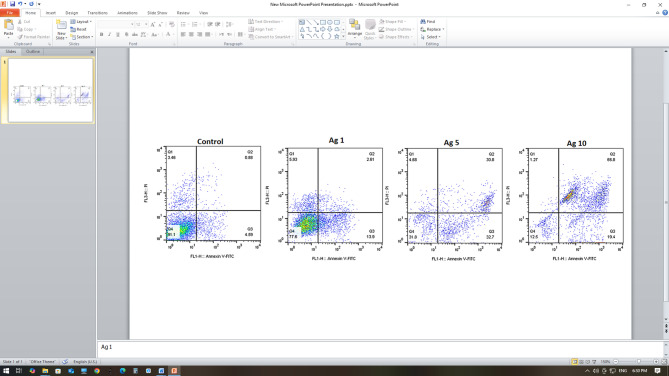
Flow cytometry results of HeLa cells treated with varying concentrations of AgNPs.

Flow cytometry analysis with Annexin V and PI staining revealed that HeLa cells underwent apoptosis in both early and late stages. The quadrants in Fig. 1 are defined as follows: Q1: necrosis, Q2: late apoptosis, Q3: early apoptosis, and Q4: live cells. The proportion of Early apoptotic cells was 13.9% at 1 µg/mL AgNPs, 32.7% at 5 µg/mL AgNPs, and 19.4% at 10 µg/mL AgNPs (Fig. 1). The 5 µg/mL AgNP treatment resulted in the highest level of early apoptosis and the lowest levels of late apoptosis and necrosis after 48 h of treatment.

**Fig. 2:**
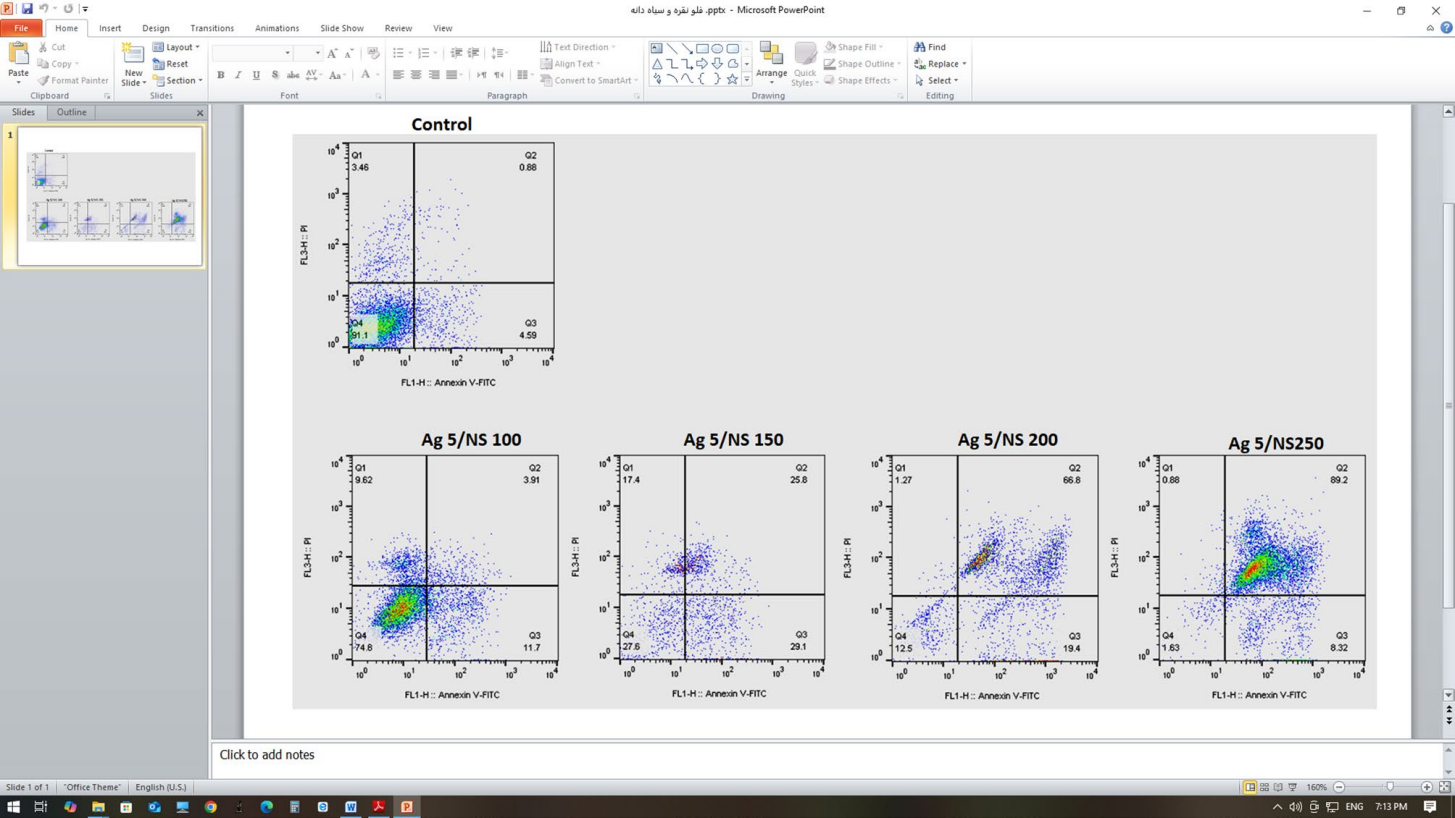
Flow cytometry results of HeLa cells treated with 5 µg/mL silver nanoparticles with varying concentrations of Nigella sativa oil.

The early apoptosis rates in the 5Ag/100 NS µg/mL, 5Ag/150 NS µg/mL, 5Ag/200 NS µg/mL, and 5Ag/250 NS µg/mL groups were 11.7, 29.1, 19.4, and 8.32, respectively (Fig. 2). The results of treatment with the optimal dose of AgNPs (5 µg/mL) combined with different concentrations of NS showed that the 5Ag/150 NS µg/mL group had the highest early apoptosis rate and the lowest secondary apoptosis and necrosis compared to the control group.

### Cell viability (MTT) assay in control and treatment groups

**Fig. 3:**
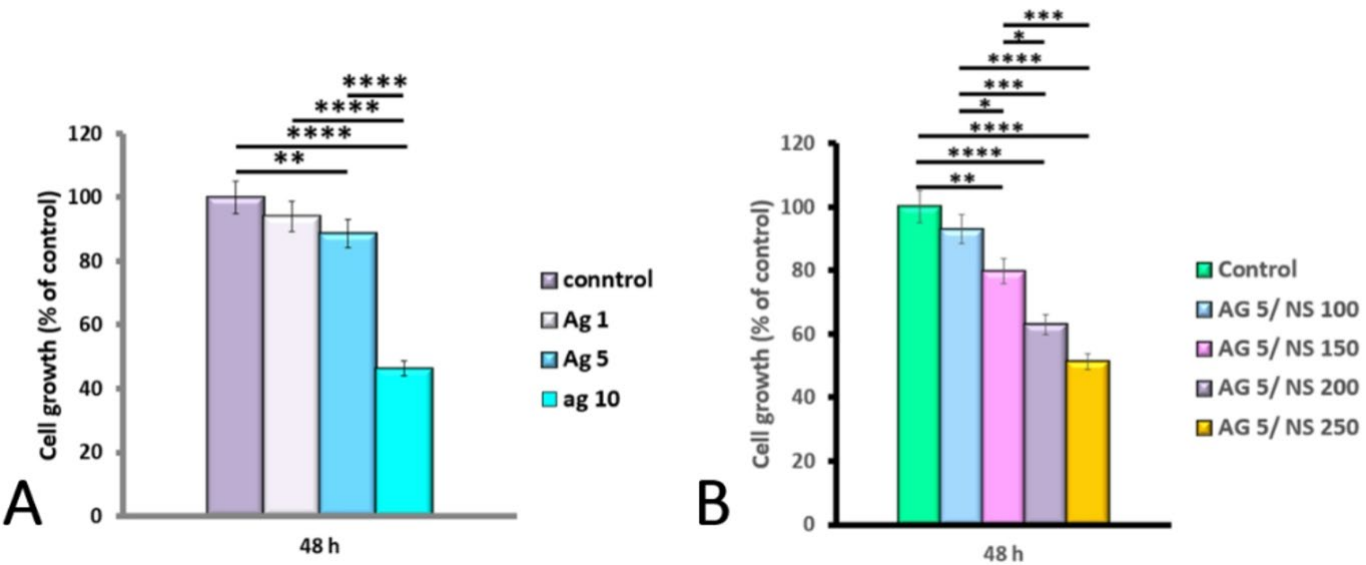
(**A**) Comparison of cell viability in HeLa cells treated with varying concentrations of AgNPs after 48 h (*P < 0.05, **P < 0.01, ***P < 0.001, ****P < 0.0001). (**B**)Comparison of cell viability in HeLa cells treated with different concentrations of 5 µg/mL AgNPs combined with NS at 100–250 µg/mL.(*P < 0.05, **P < 0.01, ***P < 0.001, ****P < 0.0001).

The cytotoxic effect of various concentrations of AgNPs (1, 5, and 10 µg/mL) and of the ideal dose of AgNPs combined with varying concentrations of NS (100, 150, 200, and 250 µg/mL) on HeLa cells was examined using the MTT assay after 48 h. Yellow tetrazolium salt is reduced to purple formazan crystals by dehydrogenase enzymes in the mitochondrial components of viable cells. Results indicated a dose-dependent decrease in cell viability in treated samples compared to the control. As shown in Fig. 3_A, the highest apoptosis (high early apoptosis and minimal late apoptosis and necrosis) was found at 5 µg/mL AgNPs.

The MTT assay confirmed the dose-dependent cytotoxicity observed in flow cytometry. Treatment with the combination of 5 µg/mL AgNPs and varying concentrations of NS revealed that the 5 Ag /150 NS µg/mL group exhibited the most favorable apoptotic profile, characterized by the highest reduction in cell viability, extensive early apoptosis, and minimal late apoptosis and necrosis (Fig. 3B). While all other combination groups (5Ag/100, 5Ag/200, 5Ag/250 NS µg/mL) also showed significant reductions in cell viability compared to the control (*p* < 0.05), the effects were less optimal. Specifically, higher NS concentrations (200 and 250 µg/mL) in combination with AgNPs led to increased levels of late apoptosis and necrosis. Consequently, the 5Ag/150 NS µg/mL combination was identified as the most effective and selective treatment condition.

### Acridine Orange/Ethidium bromide staining

**Fig. 4:**
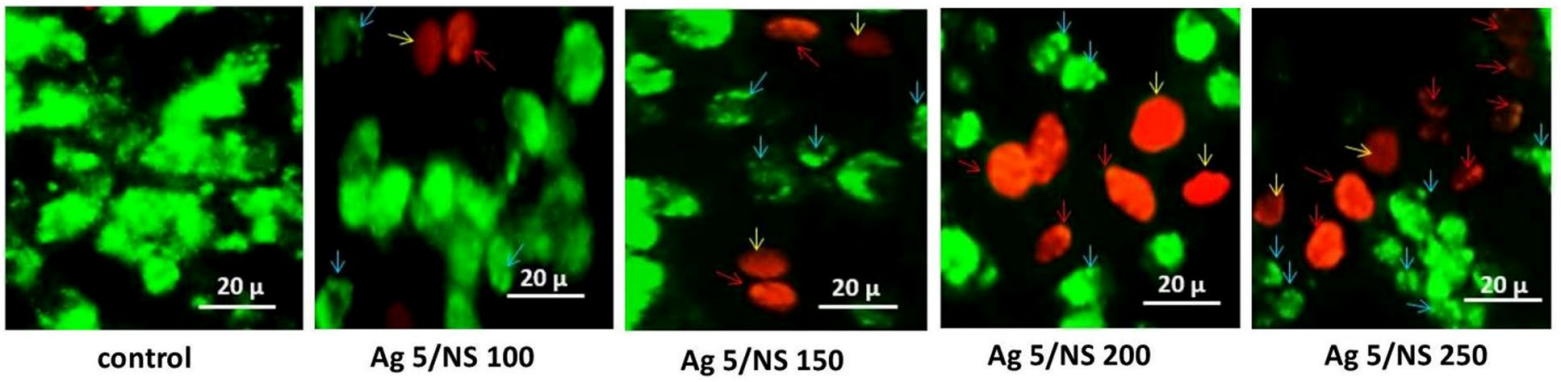
Fluorescence microscopy images of HeLa cells stained with acridine orange/ethidium bromide.

Fluorescence microscopy using acridine orange and ethidium bromide staining confirmed the induction of apoptosis in treated HeLa cells. Early apoptotic cells exhibited green fluorescence condensed or fragmented chromatin, while late apoptotic cells appeared orange due to the uptake of ethidium bromide. Necrotic cells also stained orange but lacked the chromatin condensation seen in apoptosis (Fig. 4).

**Fig. 5:**
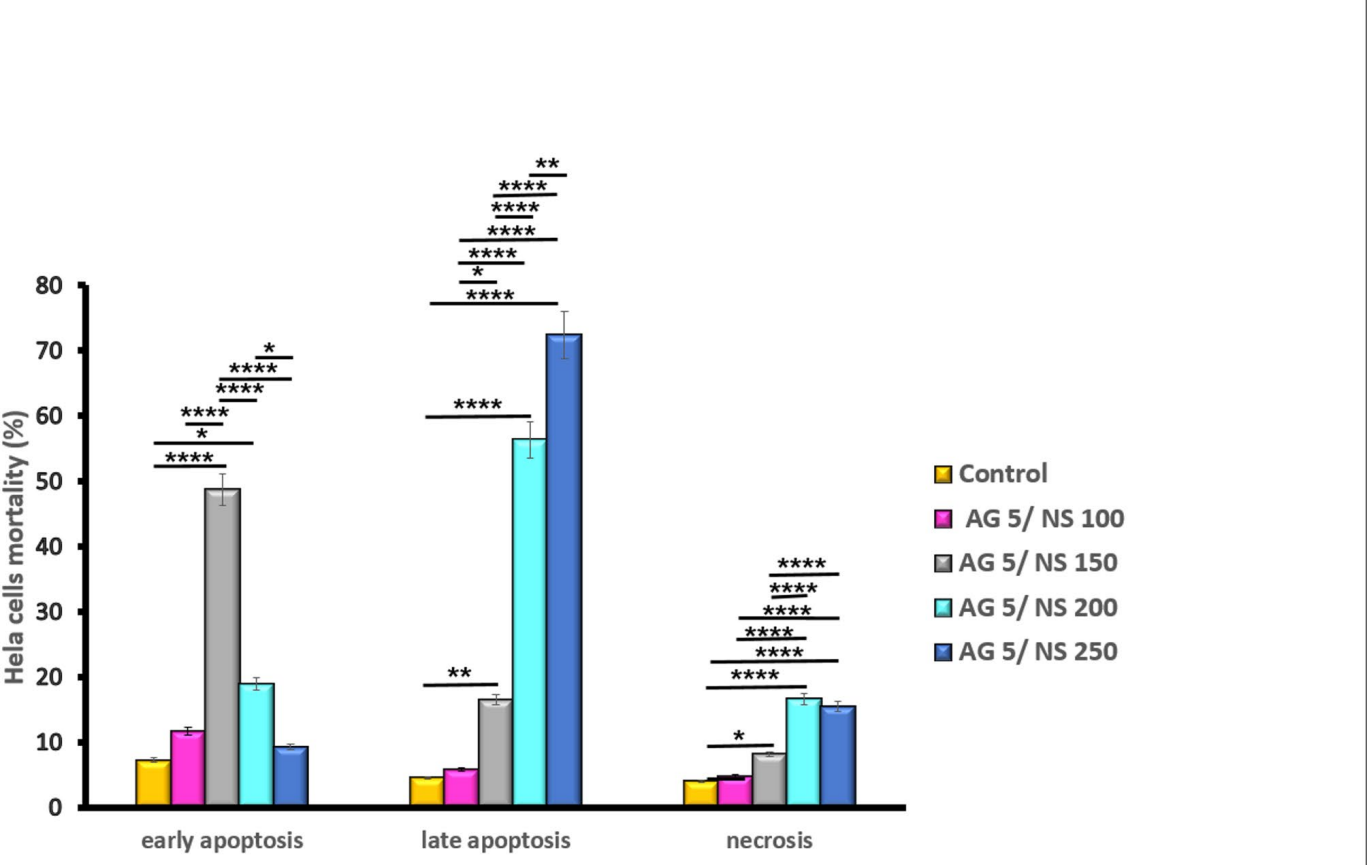
Percentage of cell death types (early apoptosis, late apoptosis, and necrosis) in control and treated HeLa cells (*P < 0.05, **P < 0.01, ***P < 0.001, ****P < 0.0001).

The comparison between groups revealed that the early apoptosis rate was significantly higher in cells treated with the combination of 5 µg/mL AgNPs and 150 µg/mL NS. Additionally, the levels of late apoptosis and necrosis were more pronounced in the groups treated with 200 µg/mL or 250 µg/mL NS in combination with 5 µg/mL AgNPs compared to the other groups (*p* < 0.0001). Significant differences in early and late apoptosis were observed between the treated and control groups (Fig. 5). Representative images from acridine orange/ethidium bromide staining assessing cell death in HeLa cells treated with 5 µg/mL AgNPs and varying concentrations of NS after 48 h. The blue arrow indicates an early apoptotic cell, the red arrow indicates a late apoptotic cell, and the yellow arrow indicates a necrotic cell.

### Expression assay

**Fig. 6:**
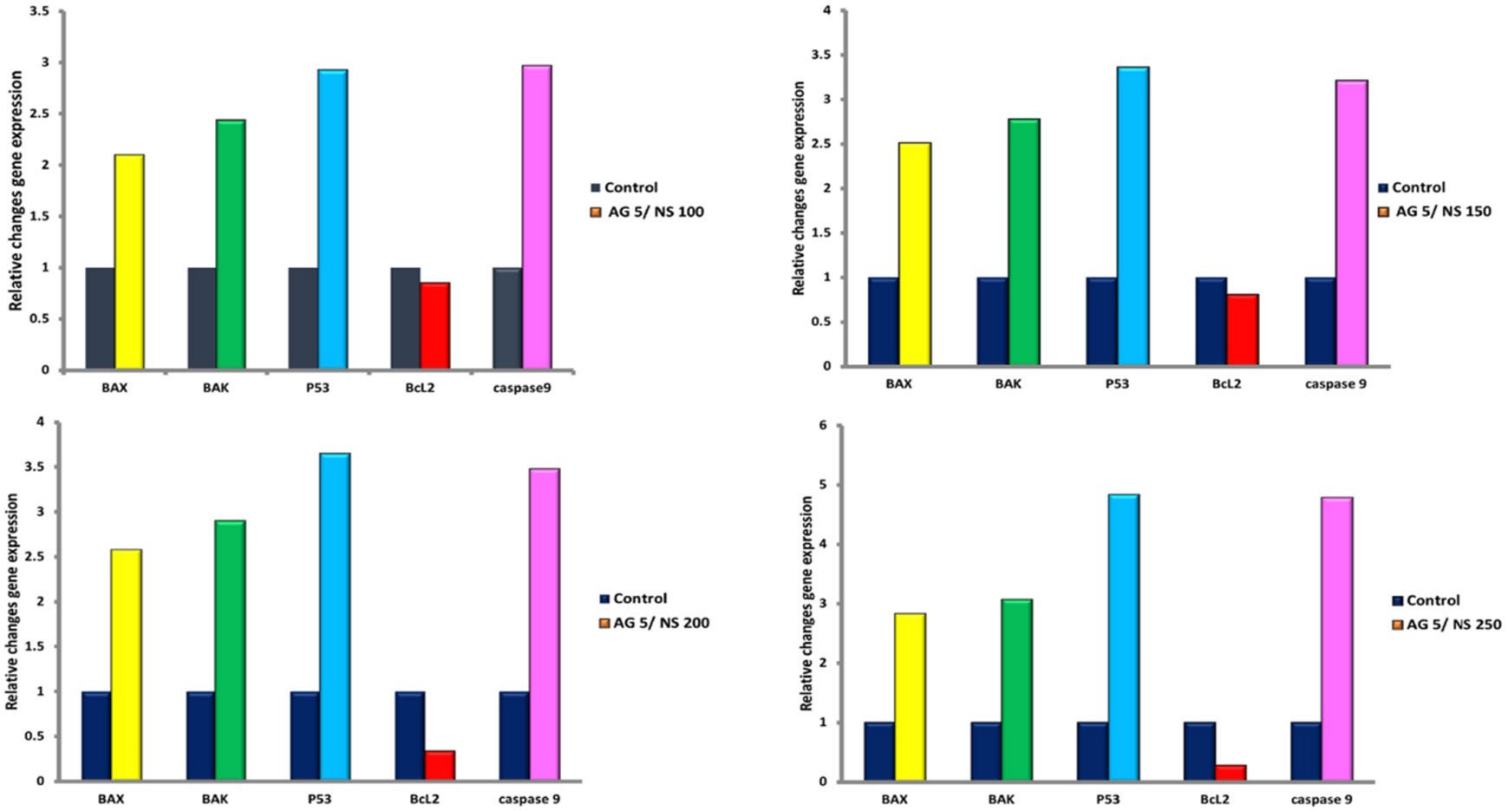
Dose-dependent modulation of apoptotic gene expression in HeLa cells treated with combined (AgNPs, 5 µg/mL) and (NS, 100–250 µg/mL). Data represent fold-change in mRNA levels of pro-apoptotic genes (*BAX*, *BAK*, *P53*, CASPASE-9) and anti-apoptotic BCL-2 relative to untreated controls (set as 1). Note the progressive upregulation of pro-apoptotic genes and suppression of BCL-2 with increasing NS concentrations, peaking at Ag 5/NS 250 µg/mL (*P53*: 4.84-fold; CASPASE-9: 4.79-fold; BCL-2: 0.28-fold).

Real-time PCR analysis was performed to quantify the mRNA expression levels of apoptosis-related genes in HeLa cells treated with AgNPs and NS. The results showed a dose-dependent upregulation of the pro-apoptotic genes BAX, BAK, P53, and CASPASE-9 genes, with the highest levels observed in the Ag5/NS200 and Ag5/NS250 groups. Significant upregulation (*p* < 0.001) was observed for P53 (3.3-fold) and Caspase-9 (3.2-fold) in cells treated with the 5 µg/mL AgNPs / 150 µg/mL NS combination. The NS group 150-treated group compared to controls(Fig. 6).

## Discussion

Our key findings demonstrate that the combination of biosynthesized AgNPs and NS exhibits a potent, synergistic cytotoxic effect on HeLa cervical cancer cells, primarily by inducing programmed cell death. The optimal therapeutic effect was achieved with a specific combination of 5 µg/mL AgNPs and 150 µg/mL NS, which induced the highest rate of early apoptosis while minimizing late apoptosis and necrosis, as confirmed by flow cytometry, fluorescence microscopy, and MTT viability assays. At the transcriptional level, this enhanced cytotoxic activity was associated with a significant dose-dependent upregulation in the mRNA of key pro-apoptotic genes (BAX, BAK, P53, CASPASE-9) and a concurrent suppression of the anti-apoptotic BCL-2 mRNA. These gene expression patterns suggest a potential mechanistic basis for the observed apoptosis; however, confirmation at the protein level is required to fully establish this pathway (as noted in the Study Limitations).

### Molecular mechanisms of synergy

The upregulation of pro-apoptotic genes (*Bax*, *Bak*, *p53*, *caspase-9*) and suppression of anti-apoptotic *Bcl-2* observed in this study corroborate prior findings on the NS role in epigenetic regulation. As highlighted by Khan et al. (2011)^9^, (TQ), the primary bioactive component of NS(identified via GC-MS in our study), inhibits the trimeric epigenetic complex UHRF1/DNMT1/HDAC1, reactivating tumor suppressor genes and promoting apoptosis. This aligns with our results showing p53 upregulation, a critical tumor suppressor gene whose activation by TQ has been extensively documented in colorectal and breast cancer models^10,11^. Similarly, AgNPs are known to disrupt mitochondrial function, potentially leading to oxidative stress and DNA fragmentation. The combination likely accelerates apoptotic signaling by concurrently destabilizing cancer cell survival mechanisms (e.g., via *Bcl-2* downregulation) and activating executioner caspases. Notably, the caspase-9 activation observed here mirrors studies where TQ induced caspase-dependent apoptosis in HL-60 leukemia and HeLa cells^12,13^, underscoring the conserved role of caspases across cancer types^12,13^.

This biphasic activity—acting as a pro-oxidant in cancer cells and an antioxidant in normal cells—is consistent with the findings of Shafi et al.^13^, who reported that NS reduced renal toxicity while enhancing the efficacy of chemotherapeutic agents. This is supported by existing literature^14^ indicating that NS sustains antioxidant enzymes like glutathione peroxidase and catalase. Clinical Implications and Novelty Cervical cancer remains a significant killer in the developing world, where advanced treatment is not widely available.

Our results are consistent with previous studies, which demonstrate that NS can sensitize cancer cells to apoptosis—a key characteristic of epigenetic modulators like TQ. Moreover, the observed synergy between AgNPs and NS is aligned with the recent trends in nanomedicine, whereby natural agents enhance nanoparticle efficacy while minimizing toxicity. For instance, TQ’s suppression of NF-κB and STAT3 pathways.

While promising, this work is limited to in vitro models. Follow-up studies should replicate these findings in vivo, in orthotopic cervical cancer models, to ascertain pharmacokinetics and systemic toxicity. Preclinical studies, such as the work of Ait Mbarek et al.^15^, which demonstrated metastasis suppression of NS in murine models, provide a methodological exemplar for future in vivo research. Additionally, exploration of the cross-talk between NS and AgNPs in modulating epigenetic regulators (e.g., HDAC1, UHRF1) can provide additional mechanistic insights. Long-term studies are also necessary to determine resistance development and immune modulation effects, particularly given the review’s emphasis on the immunostimulatory properties of NS^16^.

The intrinsic apoptotic pathway, initiated by the translocation of pro-apoptotic proteins like Bax and Bak to the mitochondria, is a well-established mechanism for triggering caspase activation and cell death^17,18^.

In late-stage apoptosis, the expression of the BCL2 gene decreases, indicating a shift from cell survival to apoptosis.

The progression to late-stage apoptosis is typically driven by sustained p53 activity and profound BCL-2 suppression, which reinforces the cell’s commitment to death^19,20^.

Research on patients with traumatic brachial plexus injury revealed that while Bcl-2 expression is elevated in the early phase (inhibiting apoptosis), Bax expression significantly increases in the late phase, leading to apoptosis. This late-stage upregulation of Bax is associated with p53 activation and the induction of Bax expression, promoting enhanced cell death. Thus, Bax is involved in the early stages of apoptosis and also plays a critical role in sustaining apoptosis during later phases^20^. Studies indicate that BAK1 plays a pivotal role in the intrinsic apoptotic pathway, and its expression increases in conditions associated with enhanced apoptosis, such as age-related hearing impairment (ARHI). The BAK1-to-BCL2 ratio (anti-apoptotic gene) rises significantly, reflecting increased apoptotic activity during late stages.

This suggests that BAK1 not only contributes to early apoptotic signaling but also helps drive apoptosis progression when cellular damage persists, further highlighting its importance in regulated cell death mechanisms^21^.

Studies show that caspase-9 is activated in the early stages, during the progression of apoptosis, and in the final stages, and helps enhance the induction of apoptosis (by activating caspase-8)^22^.

The overexpression of caspase-9 demonstrates its critical role in promoting cell death^23^. Caspase-9 activity is essential for initiating the apoptotic cascade. Its activation can occur through multiple mechanisms, including both apoptosome-dependent and apoptosome-independent pathways, as well as through cleavage of various substrates, enabling it to eliminate cellular components during apoptosis^23–25^. The gene expression results in this study confirm the apoptotic roles of BAX, BAK, p53, caspase-9, and Bcl-2 in late-stage apoptosis at concentrations of Ag 5/NS 200 and Ag 5/NS 250. Aligning with the growing interest in NS for nanomedicine, our study, alongside recent work with NS-gold nanoparticles^26^, confirms the plant’s synergistic potential with metals. A key difference emerges in the mechanism: while the prior study reported inhibition of autophagy, our AgNP-NS combination predominantly amplified the apoptotic pathway in HeLa cells, highlighting how the choice of nanoparticle material can influence the cellular response.

Looking forward, the translational potential of this combination could be explored through the development of a localized intravaginal delivery system, such as a gel or cream containing the optimal AgNP-NS formulation. This approach could be investigated as an adjunct therapy to enhance tumor cell apoptosis while potentially minimizing systemic exposure and side effects.

### Limitations of the study

This study has several limitations that should be considered when interpreting the results. Firstly, our mechanistic insights are based solely on mRNA expression data; protein-level validation (e.g., via Western blot for Bax, Bcl-2, and cleaved caspases) is necessary to confirm the activation of these apoptotic pathways. The characterization of the AgNPs was also limited to SEM and FTIR. A more comprehensive analysis, including dynamic light scattering and zeta potential, would provide a better understanding of their stability and behavior in a physiological environment. Critically, we did not directly confirm cellular uptake using techniques like TEM or ICP-MS, nor did we include a vehicle control for the ethanol solvent used in the NS extract preparation, leaving a potential, though likely minor, confounding factor unaddressed.

Furthermore, the experimental design could be strengthened in future work. The study was conducted at a single 48-hour time point, and a detailed time-course analysis would elucidate the kinetics of the apoptotic response. We also did not establish formal dose-response curves or IC₅₀ values, which are needed to precisely quantify the potency and synergistic interaction of the agents. While the consistent results across multiple assays provide strong internal validation, the absence of a known apoptosis-inducing positive control remains a limitation for benchmarking our system. Finally, while a plausible hypothesis, the proposed role of reactive oxygen species (ROS) modulation in the synergy was not directly measured and requires future investigation through dedicated assays.

## Conclusion

The integration of AgNPs and NS represents a novel multifaceted strategy for inducing apoptosis in cervical cancer cells in vitro. By combining AgNPs’ direct cytotoxic effects with NS’s epigenetic and antioxidant properties, this strategy enhances apoptotic induction. These findings contribute to the growing body of evidence supporting plant-nanoparticle synergies as a frontier in oncology. Further in vivo and translational research is warranted to validate the therapeutic potential of this combination.

## Supplementary Information

Below is the link to the electronic supplementary material.

Supplementary Material 1

## Data Availability

All data generated or analyzed during this study are included in this published article and its supplementary information files.
