# Supplementary material for: Silver nanoparticles combine with Nigella sativa oil to potentiate apoptosis in cervical cancer
Source: Sci Rep. 2026 Apr 7;16:11446. doi: 10.1038/s41598-026-36082-4 (PMC13056924; doi:10.1038/s41598-026-36082-4)
Supplement: Supplementary file 1 — Supplementary Material 1 [file 41598_2026_36082_MOESM1_ESM.docx]

**Supplementary Table 1.** Primer sequences used for real-time PCR.

| Number Primer N | Sequence 5' 3' (10-50bp) | Length | Annealing (C°) |
| --- | --- | --- | --- |
| 1 H-BAX-F | CAA ACT GGT GCT CAA GGC | 18 | 60 |
| 2 H-BAX-R | CAC AAA GAT GGT CAC GGT C | 19 | 60 |
| 3 H-P53-F | GGA GTA TTT GGA TGA CAG AAA C | 22 | 60 |
| 4 H- P53-R | GAT TAC CAC TGG AGT CTT C | 19 | 60 |
| 5 BCL2-F | GAT AAC GGA GGC TGG GAT G | 19 | 60 |
| 6 BCL2-R | CAG GAG AAA TCA AAC AGA GGC | 19 | 60 |
| 7 BAK-F | TGA AAA ATG GCT TCG GGG CAA | 21 | 60 |
| 8 BAK-R | TCA TGA TTT GAA GAA TCT TCG T | 22 | 60 |
| 9 Caspase 3-F | AAG CCG AAA CTC TTC ATC AT | 20 | 60 |
| 10 caspase 3-R | CAC TCC CAG TCA TTC CTT TA | 20 | 60 |
| 11 GAPDH-F | ACATCATCCCTGCCTCTACTG | 21 | 60 |
| 12 GAPDH-R | CCTG CTTCACCA CCTTCTTG | 22 | 60 |

**Supplementary Table 2.** Major compounds identified in the alcoholic extract of Nigella sativa oil in Gas chromatography-mass spectrometry (GC/MS) Analysis

| No | RT | % | Components | KI | Lipid Name |
| --- | --- | --- | --- | --- | --- |
| 1 | 4.50 | 1.27 | α-Thujene | 931 | MH |
| 2 | 4.68 | 0.26 | α-Pinene | 941 | MH |
| 3 | 5.40 | 0.10 | Sabinene | 981 | MH |
| 4 | 5.53 | 0.33 | β-Pinene | 988 | MH |
| 5 | 6.22 | 0.06 | α-Terpinene | 1027 | MH |
| 6 | 6.40 | 3.23 | o-Cymene | 1037 | MH |
| 7 | 6.97 | 0.29 | γ-Terpinene | 1068 | MH |
| 8 | 8.02 | 0.34 | trans-4-Methoxythujane | 1129 | MO |
| 9 | 9.44 | 0.07 | 3-p-Menthen-7-al | 1216 | MO |
| 10 | 11.67 | 0.08 | α-Longipinene | 1368 | SH |
| 11 | 12.55 | 0.64 | Longifolene | 1434 | SH |
| 12 | 16.13 | 1.23 | Tetradecanoic acid, methyl ester | 1731 | 14:0 |
| 13 | 17.21 | 0.28 | Pentadecanoic acid methyl ester | 1831 | 15:0 |
| 14 | 18.05 | 1.49 | Palmitoleic acid, methyl ester | 1912 | 16:1 (n-7) |
| 15 | 18.15 | 0.04 | 9Z-Hexadecenoic acid, methyl ester | 1922 | 16:1 (n-7) |
| 16 | 18.29 | 17.82 | Hexadecanoic acid, methyl ester | 1931 | 16:0 |
| 17 | 19.02 | 0.41 | 15-methyl-Hexadecanoic acid methyl ester | 2005 | 17:0 |
| 18 | 19.24 | 0.36 | Heptadecanoic acid, methyl ester | 2016 | 17:0 |
| 19 | 19.95 | 36.17 | Linoleic acid, methyl ester | 2054 | 18:2 (n-6) |
| 20 | 20.17 | 7.57 | 9-Octadecenoic acid, methyl ester | 2066 | 18:1 (n-9) |
| 21 | 20.26 | 8.42 | Stearic acid, methyl ester | 2071 | 18:0 |
| 22 | 20.87 | 0.12 | cis-10-Nonadecenoic acid, methyl ester | 2204 | 19:0 |
| 23 | 21.75 | 10.91 | 11Z, 14Z-Eicosadienoic acid, methyl ester | 2255 | 20:2 (n-6) |
| 24 | 21.94 | 1.22 | Eicosanoic acid, methyl ester | 2267 | 20:0 |
| 25 | 23.57 | 0.15 | Docosanoic acid, methyl ester | 2468 | 22:0 |
| 26 | 25.21 | 0.14 | Tetracosanoic acid, methyl ester | 2664 | 24:0 |
| Total Identified | 92.97% |  |  |  |  |

MH: Monoterpene Hydrocarbons, MO: Oxygenated Monoterpenes, SH: Sesquiterpene Hydrocarbons, SO: Oxygenated Sesquiterpenes

**Supplementary Table 3.** Interpretation of FTIR results for silver nanoparticles.

| Functional Groups | Wavenumber (cm-1) |
| --- | --- |
| OH | 3751 |
| OH | 3441 |
| C=C | 2915 |
| Atmospheric CO2 | 2361 |
| Silver concentration | 1629 |
| Carboxylate | 1372 |
| C-N | 1266 |
| C-N-C | 997 |
| Ag-O-Ag bridges | 875 |
| CCOOAg | 669 |
| N2O | 393 |


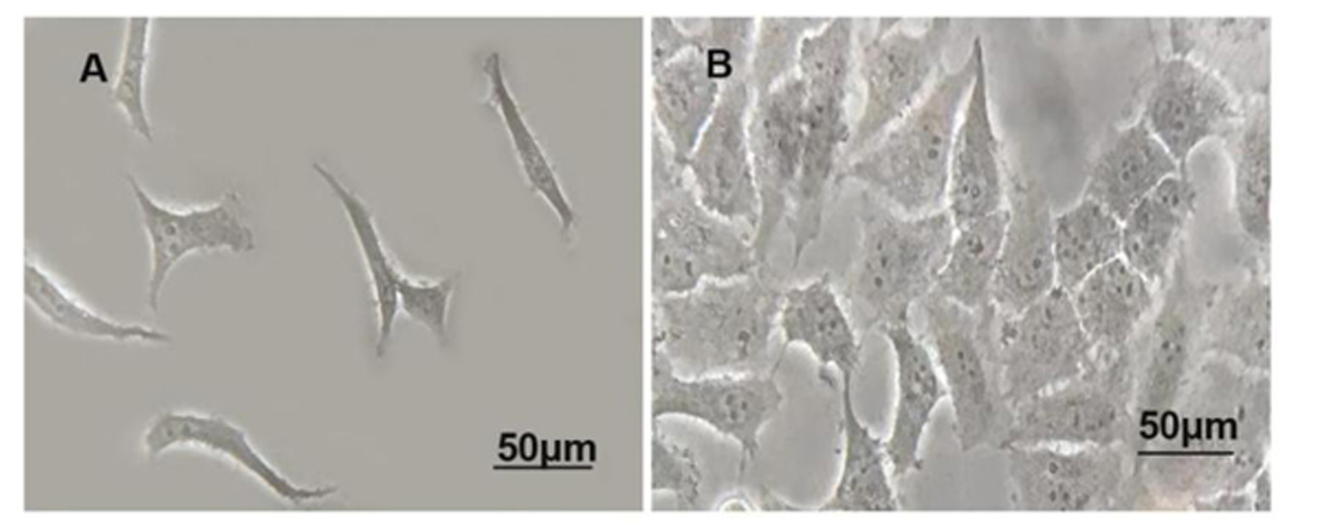


**Supplementary Figure 1.** Morphology of HeLa cells (A) after 1 d and (B) 5 d.


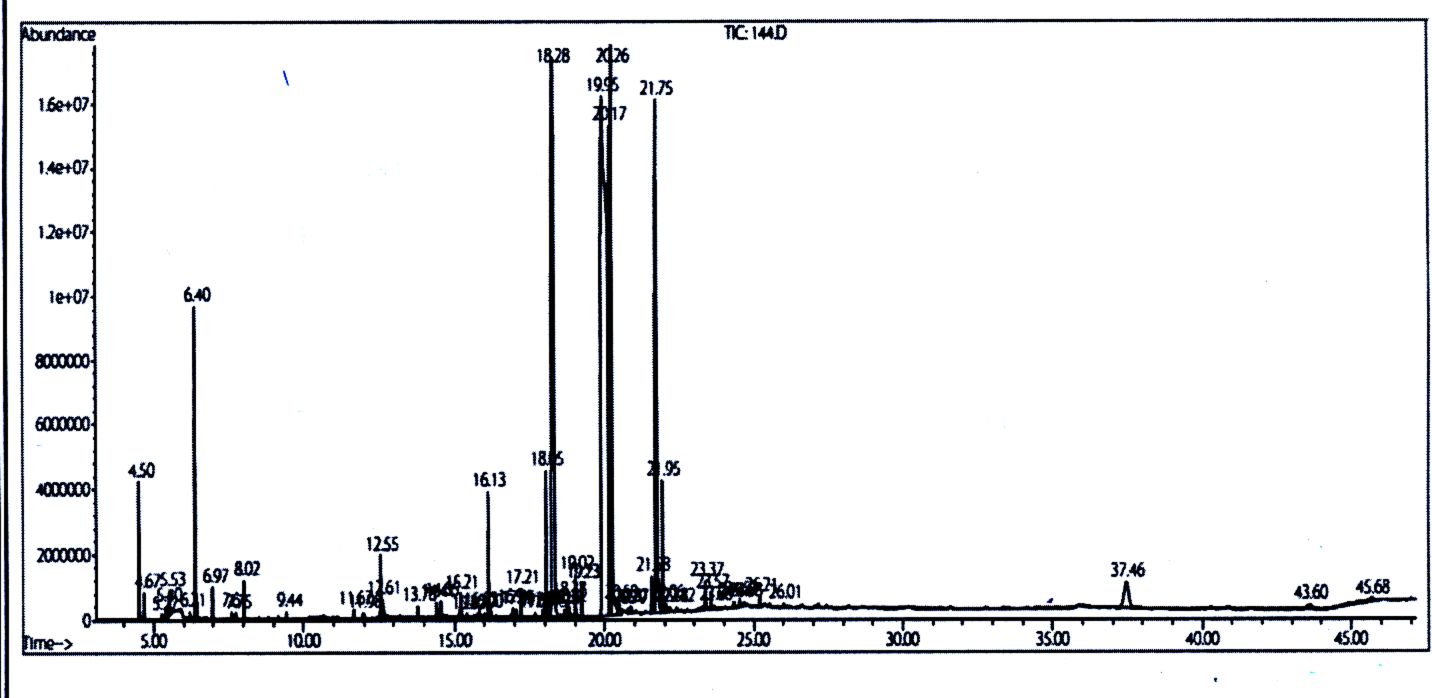


**Supplementary Figure 2.** GC-MS chromatogram of Nigella sativa extract.


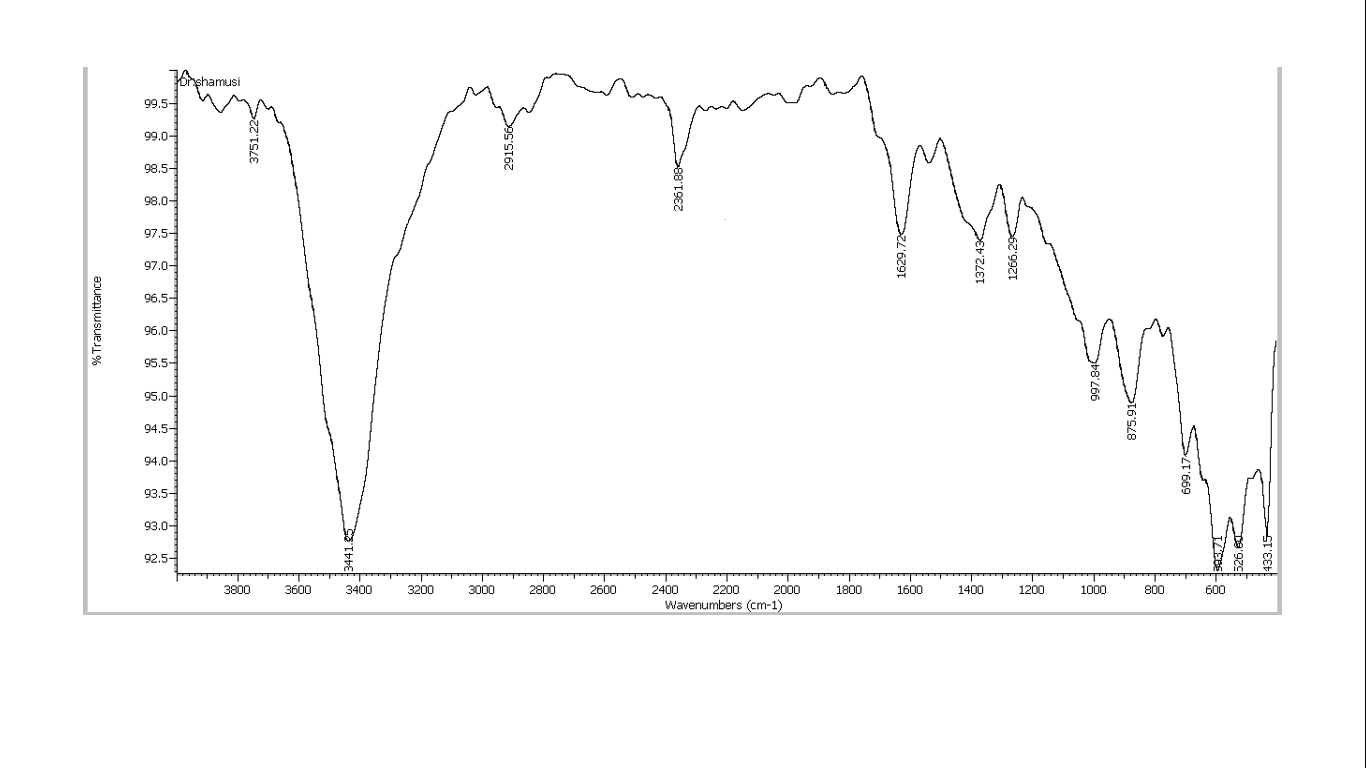


**Supplementary Figure 3.** FTIR spectrum of silver nanoparticles.


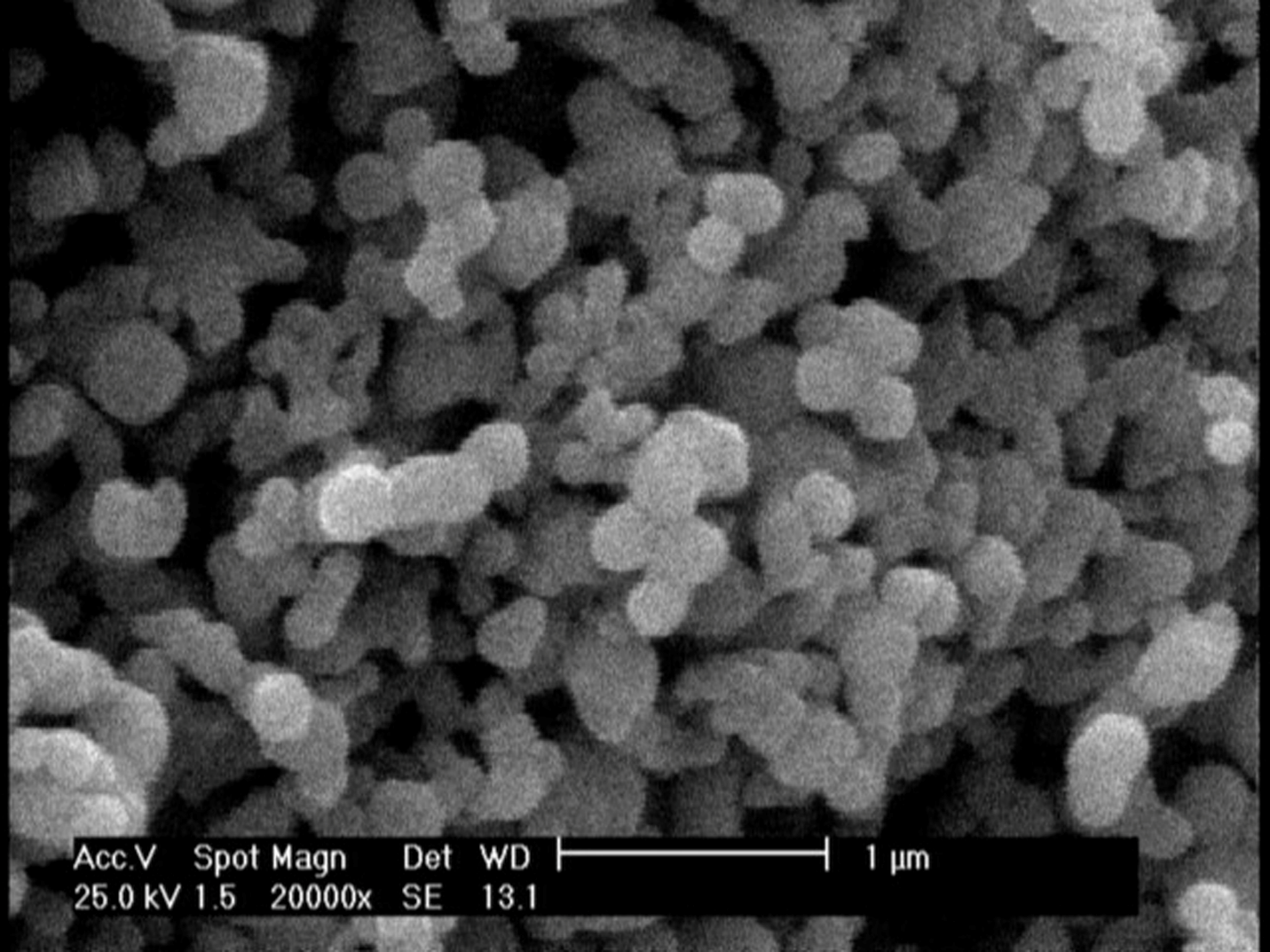


**Supplementary Figure 4.** SEM images of chemically synthesized silver nanoparticles. The average particle diameter is approximately 100 nm.
